# The Relationship between Age and Illness Duration in Chronic Fatigue Syndrome

**DOI:** 10.3390/diagnostics6020016

**Published:** 2016-04-22

**Authors:** Elizabeth Kidd, Abigail Brown, Stephanie McManimen, Leonard A. Jason, Julia L. Newton, Elin Bolle Strand

**Affiliations:** 1 Center for Community Research, DePaul University, 990 W. Fullerton Ave. Suite 3100, Chicago, IL 60614, USA; ekidd3118@gmail.com (E.K.); abrown57@depaul.edu (A.B.); smcmanim@depaul.edu (S.M.); 2 Clinical Medicine, Newcastle University, Newcastle NE2 4HH, England; julia.newton@newcastle.ac.uk; 3 Division of Medicine, Oslo University Hospital, 0450 Oslo, Norway; lbstr@ous-hf.no

**Keywords:** chronic fatigue syndrome, illness duration, age

## Abstract

Chronic fatigue syndrome (CFS) is a debilitating illness, but it is unclear if patient age and illness duration might affect symptoms and functioning of patients. In the current study, participants were categorized into four groups based upon age (under or over age 55) and illness duration (more or less than 10 years). The groups were compared on functioning and symptoms. Findings indicated that those who were older with a longer illness duration had significantly higher levels of mental health functioning than those who were younger with a shorter or longer illness duration and the older group with a shorter illness duration. The results suggest that older patients with an illness duration of over 10 years have significantly higher levels of mental health functioning than the three other groups. For symptoms, the younger/longer illness duration group had significantly worse immune and autonomic domains than the older/longer illness group. In addition, the younger patients with a longer illness duration displayed greater autonomic and immune symptoms in comparison to the older group with a longer illness duration. These findings suggest that both age and illness duration need to be considered when trying to understand the influence of these factors on patients.

## 1. Introduction

Chronic fatigue syndrome (CFS) is a chronic illness characterized by post-exertional malaise, cognitive dysfunction, unrefreshing sleep, and other symptoms [1]. While 88 abnormal gene expressions have been identified in patients with CFS, the precise etiology remains unclear [2]. Furthermore, research suggests that few patients completely recovery, as Cairns and Hotopf [3] found fewer than 10% of individuals return to their premorbid level of functioning. In spite of the debilitating nature of this illness, Looper and Kirmayer [4] found that patients with CFS perceived more stigma than patients with other chronic conditions such as Fibromyalgia or irritable bowel syndrome. The stigma associated with CFS creates barriers for access to care [5].

For individuals with CFS, there have been conflicting findings regarding how duration of illness affects symptoms and disability. It is possible that the early stages of this illness differ from later stages. Fennel [6] has been suggested that individuals experience a “crisis phase” shortly after becoming ill. This crisis phase results from the loss of control in their lives as well as loss of resources such as work status [7]. Fennell [6] proposed that over time, patients learn better ways of dealing with their symptoms. In support of this, Jason *et al*. [8] found that despite high levels of impairment, over time, individuals living with CFS may learn strategies to pace themselves to accommodate to their illness [8]. Jason and colleagues [8] have found that helping patients conserve appropriate energy expenditures in coordination with available energy can help patients better cope with this illness.

Still, it is unclear whether those who have been ill for longer periods have a different health status from those who have been ill for briefer periods of time. For example, several researchers [9,10] have found that a longer illness duration predicts worse outcomes. Likewise, Friedberg, Dechene, McKenzie, and Fontanetta [11] found that individuals who have had the illness for a longer period of time have significantly higher illness severity than those with shorter illness duration. Specifically, those who have had CFS for ten years or more had worse cognitive functioning than those who had been sick for seven years or less. Furthermore, van der Werf and colleagues [12] found that patients with an illness duration of two years or longer reported more concentration problems, greater fatigue, and more functional disability than patients with a shorter illness duration.

However, others have found different results. For example, Hornig *et al*. [13] found distinct alterations in plasma immune signatures among patients with CFS with an illness duration less than three years that were not present in patients with an illness duration three years or longer. More specifically, patients with CFS with a short illness duration had increased amounts of many different types of cytokines in comparison with those with a longer illness duration. In addition, Matthews and Komaroff [14] found that physical functioning tended to improve over time for many individuals with CFS. 

Still others have not found differences in those with different illness durations. For example Wilson and colleagues [15] did not find illness duration to predict patient outcome, and Hill, Tiersky, Scavalla, and Natelson [16] found that illness outcome was not predicted by length of illness. Similarly, Brown, Brown and Jason [17] found no difference in functioning between those with an illness duration of two years or longer and those with an illness duration shorter than two years. In addition, Santamarina-Perez *et al*. [18] found that cognitive functioning in patients who were sick for more than four years did not differ from patients who were sick less than one year. The reasons for these discrepant findings are unclear, but different ages of patients might have influenced these findings.

A patient’s age may contribute to differences in patient functionality and symptomatology [19]. Joyce, Hotopf, and Wessely [20] found that older age predicts a poorer CFS prognosis. In a prospective, longitudinal study, Jason *et al*. [21] found that that those that recovered over time from CFS were significantly younger than those that developed the illness. Loge, Ekeberg, and Kaasa [22] found significantly higher fatigue scores in older individuals with CFS compared to younger individuals with CFS, which was also found by Song, Jason, and Taylor [23].

The association between illness duration and patient age, with illness severity and symptomatology for individuals with CFS is unclear. Prior CFS research has focused either upon the relationship of illness duration on illness severity or the relationship of age on illness severity. However, studies have not examined the overlapping effects of both illness duration and age on patient outcomes. The current study investigated the association between illness duration and patient age on measures of functionality and symptomatology. The conflicting findings in the literature on these issues of age and duration of illness make it difficult to develop testable hypotheses in this exploratory study.

## 2. Methods

### 2.1. Participants

Participants were collected from multiple settings and geographic locations. To be eligible, individuals must have been at least 18 years of age and have a current diagnosis of ME or CFS. There was no standardized diagnostic criteria that the patients must meet to participate in the study. As described below, patients had the diagnosis confirmed by a physician for the Norway, Newcastle, and BioBank samples. However, the DePaul sample was a convenience sample of individuals with a self-reported current diagnosis of ME or CFS. Participants were recruited from multiple countries and settings to obtain a sample that would allow the findings to be generalized to the entire patient population.

#### 2.1.1. DePaul Sample

An international convenience sample of English speakers above the age of 18 was recruited. Individuals self-identified as having a ME or CFS diagnosis and were recruited from various means such as Internet forums, support group visits, and through contacting those who previously participated in DePaul studies. Participants were able to participate in the study by choosing to complete an electronic survey, a hard-copy survey, or a verbal survey over the phone. They had the option of completing the survey in person at DePaul University or at home. The first 100 participants who completed the survey were compensated with a $5.00 gift card to Amazon.com.

Demographically, the sample of 155 participants was 84.4% female and 15.6% male. Of the sample, 99.3% were identified as Caucasian and 0.7% as Asian. Of the sample, 55.8% stated that they were currently on disability, with only 13.6% of the sample working part- or full-time. As for marital status, 57.7% of the participants were married or living with someone, 22.8% were single, and 19.5% were divorced, separated, or widowed. With regard to educational level, 22.7% of the sample completed high school or had a general education diploma (GED), 38.3% attended college for at least one year, and 39.0% held a standard college degree. The mean age was 51.6 (SD 11.2).

#### 2.1.2. SolveCFS BioBank Sample

A physician-recruited sample was gathered by the Solve ME/CFS Initiative. The data originated from the SolveCFS BioBank. Participants were 18 or older and were diagnosed with ME or CFS by a licensed physician who specialized in ME or CFS. The eligible participants provided written informed consent and completed study measures by hard copy or electronically. Then, the SolveCFS Biobank data were de-identified, and the DePaul research team was given access to the data.

Demographically, the sample of 225 participants was 99.1% Caucasian, and 0.9% selected “Other” for their race. With regard to gender, 72.4% of the sample was female. Only 10.7% of the sample was working full- or part-time, with 66.2% on disability. Concerning marital status, 56.9% of the participants were married or living with someone, 26.7% were single, and 16.4% were divorced, separated, or widowed. Regarding education level, 23.6% had completed college, 44.0% had completed some college, and 32.4% had a high school degree or GED. The average age of the sample was 49.7 (SD = 12.9).

#### 2.1.3. Newcastle Sample

Individuals who were thought of having a ME or CFS diagnosis were referred to Newcastle-upon-Tyne Royal Victoria Infirmary. A physician determined a ME or CFS diagnosis by performing a complete medical history and examination. Participants who met eligibility criteria then provided written consent and completed the study measures by hard copy.

Concerning demographics, this sample of 77 participants was 98.7% Caucasian and 1.3% selected “Other” for their race. 83.1% of participants were female. Of this sample, 32.5% of participants were working either part- or full-time, and 36.4% were on disability. As for marital status, 51.9% of the participants were married or living with someone, 31.2% were single, and 16.9% were divorced, separated, or widowed. With regard to education level, 23.0% had a college degree, 28.4% had completed at least one year of college, and 28.4% had a high school degree. The average age of the sample was 45.6 (SD = 14.0).

#### 2.1.4. Norway sample 1

Participants were recruited around Oslo, Norway, upon an invitation for a randomized controlled trial of a CFS self-management program. Recruitment methods included CFS patient organizations, healthcare professionals, and a waiting list for the patient education program. Further, information regarding the study was dispersed through brochures and posted on the Oslo University Hospital website. 

To be eligible, participants needed to be 18 or older and be diagnosed with CFS by a physician. The participants were required to physically attend the self-management program. Participants provided consent that allowed physicians to confirm their CFS diagnosis. The Regional Committee for Medical Research Ethics and the Privacy Ombudsman for Research as Oslo University Hospital gave approval for the study.

This sample of 151 participants was 87.3% female and 12.7% male. Almost all participants were Caucasian (99.3%); one participant selected “Other” when asked about race. Only 9.3% of participants were working, while 82.8% were on disability. As for marital status, 60.3% of the participants were married or living with someone, 25.8% were single, and 13.9% were divorced, separated, or widowed. Concerning education, 49.0% had a high school degree or GED, 39.6% had completed at least on year of college, and 11.4% held a standard college degree. The mean age of the sample was 43.4 years (SD = 11.7).

#### 2.1.5. Norway Sample 2

A severely ill sample was recruited from an inpatient medical ward and a ME/CFS outpatient clinic. Individuals were between 18 and 65 years old and were able to read and write Norwegian. Individuals who were suspected to have ME or CFS were assessed by medical professionals to rule out exclusionary medical and psychiatric conditions. Participants gave written informed consent and completed the study measures by hard copy. The Privacy Ombudsman for research at Oslo University Hospital granted approval for the study.

This sample of 44 participants was 81.8% female and 18.2% male. The majority of the sample identified as Caucasian (93.0%), but 7.0% as “Other”. Most participants (81.8%) were on disability, while 13.6% were working. As for marital status, 52.3% of the participants were married or living with someone, 43.2% were single, and 4.5% were divorced, separated, or widowed. Concerning education, 70.4% had a high school degree or GED and 29.5% held a standard college degree. The mean age of the sample was 34.9 years (SD = 11.6).

### 2.2. Procedure

Participants (*N* = 652) answered sociodemographic questions regarding gender, race, work status, marital status, illness duration, and age. Participants then completed the DePaul Symptom Questionnaire and the Medical Outcomes Study-Short Form-36 Health Survey (SF-36).

### 2.3. Measures

#### 2.3.1. Functionality

The eight subscales of the SF-36 were used to assess functionality [24]. These subscales include physical functioning, bodily pain, mental health functioning, role emotional, social functioning, vitality, general health functioning, and role physical. Higher scores indicate better functioning with a range of 0 to 100. This scale has good discriminant validity and internal consistency based upon clinical standards [24]. The SF-36 has shown adequate psychometric properties in the measurement of functional status in a CFS sample [25].

#### 2.3.2. Symptoms

Participants completed the DePaul Symptom Questionnaire (DSQ), a self-report measure of ME and CFS symptomatology, demographics, and medical, occupational and social history [26]. This measure was developed to classify individuals by a variety of ME and CFS case definitions, but the list of 54 symptoms was based upon a revised approach to the Clinical Canadian criteria [27]. Participants rate each symptom’s frequency over the past six months on a 5-point Likert scale: 0 = none of the time, 1 = a little of the time, 2 = about half the time, 3 = most of the time, and 4 = all of the time. Likewise, participants rate each symptom’s severity over the past six months on a 5-point Likert scale: 0 = symptom not present, 1 = mild, 2 = moderate, 3 = severe, 4 = very severe. Frequency and severity scores were multiplied by 25 to create 100-point scales. The 100-point frequency and severity scores for each symptom were averaged to create one composite score per symptom. The 100-point scale was used to make results easier to understand. The scaling has no effect on the veracity of the analyses as the same results are obtained with the original 5-point scale. The DSQ has evidenced good test-retest reliability [28] and good content validity [29]. The DSQ is available in the shared library of Research Electronic Data Capture (REDCap) [30], hosted at DePaul University: https://redcap.is.depaul.edu/surveys/?s=tRxytSPVVw.

Factor analytic studies such as Jason, Sunnquist *et al.* [31], have found cognitive dysfunction, post-exertional malaise, and sleep difficulties are cardinal symptom clusters. In addition, the Canadian criteria [27] have listed Pain, Autonomic, Neuroendocrine, and Immune domains as being critical in diagnosing patients. We therefore used these findings to develop summary scores, which have also been used in prior research [21,26,32].

### 2.4. Groups

Participants were placed in one of four illness duration/age groups based upon their responses to questions regarding length of illness and age as reported on the DSQ. Based off of previous literature, an illness duration of 10 years was used as the cutoff between a shorter or longer illness duration [11]. The age cutoff was set at 55 years, which was also based off prior research [19]. It could certainly be argued that younger could mean those who are in their teenage years or before age 30. However, we used the age cutoff of 55 years based on prior research [19]. The four groups were labeled younger/shorter illness duration (*n* = 333), younger/longer illness duration (*n* = 106), older/shorter illness duration (*n* = 76), and older/longer illness duration (*n* = 135). One-way ANCOVAs, controlling for education, were run to analyze mean differences between groups on symptomatology and functionality. A Tukey *post-hoc* test was used for further interpretation of between group differences.

### 2.5. Results

#### 2.5.1. Demographics

For the overall sample, 80.6% of the participants were female, and the mean age was 47.1 (SD = 13.1). The mean illness duration was 9.1 years (SD = 7.5). Regarding ethnicity, 98.2% of the participants were White, 0.5% were Asian or Pacific Islander, and 0.8% identified as other. As for marital status, 56.9% of the participants were married or living with someone, 27.2% were single, and 15.9% were divorced, separated, or widowed. For work status, 65.1% were on disability, 13.8% of the participants were working, 12.7% were students, homemakers, or unemployed, and 8.4% were retired. For education, 36.8% had a standard college degree, 24.8% of the participants had a graduate professional degree, 21.7% had a high school diploma or GED, and 16.7% had partial college.

#### 2.5.2. Group Differences

No significant differences were found between the groups on gender or ethnicity, *p* > 0.05. However, there were significant differences between the four groups on marital status, [*X*^2^ (15, *n* = 646) = 79.2, *p* < 0.01], level of education, [*X*^2^ (9, *n* = 646) = 52.0, *p* < 0.01], and work status, [*X*^2^ (21, *n* = 651) = 155.5, *p* < 0.01]. The differences between groups for marital status and work status were expected due to age being utilized as an independent variable. Level of education was controlled for in subsequent analyses.

As shown in Table 1, the younger/shorter illness duration group (Mean = 17.9, SD = 15.5) showed statistically higher levels of vitality than the younger/longer illness duration group [(Mean = 13.2, SD = 12.3), *p* =0.033]. There was an overall effect for physical functioning [*F* (3, 619) = 3.47, *p* = 0.016], but *post-hoc* tests revealed no statistically significant between-group differences. In addition, the older/longer illness duration group (Mean = 73.8, SD = 16.7) had significantly higher levels of mental health functioning than the younger group with a short illness duration (Mean = 67.5, SD = 18.7), the younger group with a long illness duration (Mean = 67.7, SD = 17.5) and the older group with short illness duration [(Mean = 64.6, SD = 19.7), *p* = 0.002]. No significant differences were found between the groups on the other subscales-role physical, bodily pain, general health, social functioning, or role emotional.

For symptoms, the younger/longer illness duration group had significantly worse scores on the autonomic domain [(Mean = 27.9, SD = 20.8), *p =* 0.020] than the older/longer illness group (Mean = 18.3, SD = 15.4). In addition, the younger/longer illness duration group had a significantly worse scores on immune symptom domain [(Mean = 25.6, SD = 16.3), *p* = 0.039], than the older/longer illness group (Mean = 18.1, SD = 14.1). The younger/shorter illness duration group (Mean = 29.5, SD = 22.8) had significantly worse scores on the neuroendocrine domain than the older/longer illness duration group [(Mean = 21.7, SD = 20.8), *p* = 0.043]. No significant differences were found across the symptom domains of fatigue, sleep dysfunction, pain, or neurocognitive.

## 3. Discussion 

The goal of this study was to examine the effects of age and illness duration on patient functionality and symptoms. Our major finding is that both age and duration of illness needs to be taken into account when understanding the effects of these variables on functioning and symptoms. We found that the younger/shorter illness duration group showed statistically higher levels of vitality than the younger/longer illness duration group. In addition, older patients with an illness duration of over 10 years have significantly higher levels of mental health functioning than the three other groups. For symptoms, the younger/longer illness duration group had significantly worse immune and autonomic domains than the older/longer illness group. In addition, the younger patients with a longer illness duration displayed greater autonomic and immune symptoms in comparison to the older group with a longer illness duration.

Findings indicated that people with CFS who were under the age of 55 with an illness duration of fewer than ten years had greater vitality than those who were similarly aged but had been ill longer. As the illness duration increased, younger individuals’ vitality deteriorated. It is possible that individuals with CFS may lose vitality over time due to worse physical functioning as it becomes more challenging for them to do functional tasks as the illness progresses. This finding supports prior research that a longer illness duration suggests greater illness severity [11]. This difference was not found in the older patient group, which suggests the difference in vitality may be unique to younger individuals with CFS.

The older individuals with a longer illness duration indicated the best mental health functioning over the other groups. Those with a longer illness duration may have learned more effective coping skills such as planning and acceptance compared to the shorter illness duration group [17]. This supports previous findings that patients with CFS endure a “crisis” stage early in their illness [6], where they experience emotional distress and may be overwhelmed by the challenges of this debilitating illness [33].

Moreover, older individuals with a longer illness duration reported better autonomic and immune scores than young individuals with a longer illness duration. The older patient group with a long illness duration also reported better neuroendocrine scores than the young patient group with a shorter illness duration. These differences support Chou’s [19] prior research in that a patient’s age may contribute to patient symptomatology. This may help explain why older individuals experience greater levels of mental health functioning since they indicated better symptom scores than the other patient groups.

There are limitations to this study. The sample was predominately white and thus the findings may not be generalizable to a more diverse patient population. A prior study has shown that CFS does include many from African American and Latino populations [34], but more tertiary care recruitment efforts have identified less diversity among samples. Future studies need to include more diverse populations to investigate possible cultural differences.

## 4. Conclusions

These results suggest there is a meaningful relationship between age, illness duration, functionality, and symptoms in a CFS sample. These preliminary findings provide some support for the notion of a “crisis phase” of the illness [6]. In other words, the finding that patients who have been ill longer have better mental health functioning than those who are earlier in their illness. In addition, these findings suggest that individuals with CFS, who are in the early phase of their illness, might need additional supports and resources to deal with the devastating aspects of this illness. Our study helps in the understanding of functionality and symptoms as a result of illness duration and patient age, and has implications for distinguishing these groups in future research studies.

## Figures and Tables

**Table 1:** Comparing subgroups of individuals with chronic fatigue syndrome (CFS) defined by age and illness duration on mean functioning and symptomatology (*N* = 652).

Variables	Younger (<55), Illness Duration <10 Years	Younger (<55), Illness Duration >10 Years	Older (>55), Illness Duration <10 Years	Older (>55), Illness Duration >10 Years	Sig.
	(*n* = 334)	(*n* = 107)	(*n* = 76)	(*n* = 135)	
	*M* (SD)	*M* (SD)	*M* (SD)	*M* (SD)	
*SF-36 Subscales*					
Vitality	17.9 (15.5) ^a^	13.2 (12.3) ^a^	14.6 (15.4)	18.8 (16.4)	*
Physical Functioning	38.5 (23.1)	30.6 (22.8)	31.1 (22.9)	37.3 (21.4)	*
Role Physical	5.7 (17.0)	5.8 (17.5)	6.4 (11.8)	4.6 (16.2)	-
Bodily Pain	40.8 (25.5)	40.0 (25.8)	38.2 (22.8)	44.2 (21.0)	-
General Health	27.2 (16.6)	24.1 (16.3)	27.2 (16.3)	30.4 (17.4)	**-**
Social Functioning	26.9 (23.1)	22.7 (23.2)	24.7 (24.8)	31.7 (24.8)	-
Mental Health	67.5 (18.7) ^a^	67.7 (17.5) ^b^	64.6 (19.7) ^c^	73.8 (16.7) ^a,b,c^	**
Role Emotional	72.9 (40.4)	73.8 (41.8)	64.4 (43.3)	74.4 (37.7)	-
*Domain Composites*					
Fatigue/PEM	50.7 (22.8)	53.7 (23.4)	52.2 (24.4)	45.3 (24.9)	-
Sleep Dysfunction	34.8 (19.4)	36.8 (18.7)	34.8 (17.2)	30.2 (18.1)	-
Pain	28.7 (18.4)	30.8 (20.3)	29.8 (16.4)	23.7 (14.5)	-
Neurocognitive	70.3 (40.2)	63.1 (32.3)	77.0 (37.3)	62.7 (36.7)	-
Autonomic	23.9 (17.7)	27.9 (20.8) ^a^	23.9 (15.6)	18.3 (15.4) ^a^	*
Neuroendocrine	29.5 (22.8) ^a^	31.2 (20.4)	27.5 (15.0)	21.7 (20.8) ^a^	*
Immune	23.4 (16.3)	25.6 (16.3) ^a^	21.4 (13.4)	18.1 (14.1) ^a^	*

* p < 0.05, ** *p* < 0.01. Similar letters (a, b and c) across rows indicate significant differences in *post-hoc* analyses.
